# Validation of a method to assess emphysema severity by spirometry in the COPDGene study

**DOI:** 10.1186/s12931-020-01366-4

**Published:** 2020-05-01

**Authors:** Mariaelena Occhipinti, Matteo Paoletti, James D. Crapo, Barry J. Make, David A. Lynch, Vito Brusasco, Federico Lavorini, Edwin K. Silverman, Elizabeth A. Regan, Massimo Pistolesi

**Affiliations:** 1 Section of Respiratory Medicine, Department of Experimental and Clinical Medicine, University of Florence, Largo A. Brambilla 3, 50134 Florence, Italy; 2 Section of Radiology, Department of Biomedical, Experimental, and Clinical Sciences, University of Florence, Largo A. Brambilla 3, 50134 Florence, Italy; 3 Department of Medicine, National Jewish Health, 1400 Jackson St, Denver, CO 80206 USA; 4 Department of Radiology, National Jewish Health, 1400 Jackson St, Denver, CO 80206 USA; 5 Department of Experimental Medicine, University of Genoa, Via Leon Battista Alberti 2, 16132 Genoa, Italy; 6 Department of Medicine, Harvard Medical School, Brigham and Women’s Hospital, Channing Division of Network Medicine, 75 Francis St, Boston, MA 02115 USA

**Keywords:** Spirometry, Emphysema, Airway obstruction, Computed tomography, Vital capacity

## Abstract

**Background:**

Standard spirometry cannot identify the predominant mechanism underlying airflow obstruction in COPD, namely emphysema or airway disease. We aimed at validating a previously developed methodology to detect emphysema by mathematical analysis of the maximal expiratory flow-volume (MEFV) curve in standard spirometry.

**Methods:**

From the COPDGene population we selected those 5930 subjects with MEFV curve and inspiratory-expiratory CT obtained on the same day. The MEFV curve descending limb was fit real-time using forced vital capacity (FVC), peak expiratory flow, and forced expiratory flows at 25, 50 and 75% of FVC to derive an emphysema severity index (ESI), expressed as a continuous positive numeric parameter ranging from 0 to 10. According to inspiratory CT percent lung attenuation area below − 950 HU we defined three emphysema severity subgroups (%LAA_-950insp_ < 6, 6–14, ≥14). By co-registration of inspiratory-expiratory CT we quantified persistent (%pLDA) and functional (%fLDA) low-density areas as CT metrics of emphysema and airway disease, respectively.

**Results:**

ESI differentiated CT emphysema severity subgroups increasing in parallel with GOLD stages (*p* < .001), but with high variability within each stage. ESI had significantly higher correlations (*p* < .001) with emphysema than with airway disease CT metrics, explaining 67% of %pLDA variability. Conversely, standard spirometric variables (FEV_1_, FEV_1_/FVC) had significantly lower correlations than ESI with emphysema CT metrics and did not differentiate between emphysema and airways CT metrics.

**Conclusions:**

ESI adds to standard spirometry the power to discriminate whether emphysema is the predominant mechanism of airway obstruction. ESI methodology has been validated in the large multiethnic population of smokers of the COPDGene study and therefore it could be applied for clinical and research purposes in the general population of smokers, using a readily available online website.

## Background

Expiratory airflow obstruction as detected by spirometry is the hallmark of chronic obstructive pulmonary disease (COPD). In each patient the relative contribution of conductive airways narrowing and emphysematous parenchymal destruction determines the complexity and heterogeneity of the clinical presentation in COPD [1–4]. Emphysema is a major determinant of lung function decline and all-cause mortality in patients with COPD [5, 6]. Lung volumes and diffusing capacity are traditionally used to infer the presence and the severity of emphysema. However, these measurements are not always available in clinical practice, not usually included among the variables to enroll patients in large clinical and pharmacologic trials, and considered not to be essential to patient management by the 2019 Report of the Global Initiative for Chronic Obstructive Lung Disease (*https://goldcopd.org/*).

Recently the application of computed tomography (CT) to the study of COPD has provided information on the pathological changes occurring in the disease [7, 8]. Bronchial wall thickening, gas trapping, and parenchymal destruction are qualitatively evaluated and quantitatively assessed by dedicated software [9–12]. Identification of the prevailing mechanism for expiratory airflow obstruction, i.e. airways disease vs. parenchymal destruction, can be obtained by CT analyses, such as parametric response maps from co-registration of inspiratory and expiratory scans [13] and disease probability measure maps [14]. Quantitative evaluation of CT scans has been introduced in large-scale COPD population studies [15, 16]. However, the high prevalence of COPD in the general population [17], the relative limited availability of CT and its intrinsic use of ionizing radiation limit the use of CT imaging for the diagnosis and treatment of COPD in clinical routine as well as in large-scale clinical, epidemiologic, and pharmacologic trials. Furthermore, CT metrics do offer a quantitative evaluation of the extent of lung pathology but no information on the mechanism of airflow limitation.

A model based on the mathematical fitting of the descending limb of the maximal expiratory flow-volume curve (MEFV) in standard spirometry can provide a functional emphysema severity index (ESI) that strongly correlates with the extent of emphysema on CT-based radiomics, as demonstrated in a previous study conducted in a small cohort of Caucasian patients with COPD [18].

The aim of the present study was to validate by CT metrics the ESI methodology for the assessment of emphysema severity by standard spirometry in the large multiethnic population of US subjects enrolled in the COPDGene study, in order to demonstrate the generalizability of the method for pharmacologic trials and its utility in clinical care of former and current smokers.

## Methods

COPDGene is a multicenter study designed to identify genetic factors, to characterize CT subtypes, and determinants of progression. Study details have been previously published [16]. Institutional review boards approved the study across the 21 US participating Clinical Centers between January 2008 and June 2011 and all participants provided written informed consent. COPDGene included current and former smokers aged 45–80 years, either non-Hispanic whites or non-Hispanic African Americans. The study included smoker controls (GOLD 0), GOLD 1–4 and PRISm (Preserved Ratio Impaired Spirometry, i.e. FEV_1_/FVC ≥ 0.7 and post-bronchodilator FEV_1_ < 80% of predicted) subjects for a total of 10,371 subjects with at least 10 pack-years smoking history [16]. All participants underwent spirometry and inspiratory-expiratory CT.

### Spirometry

All subjects underwent pre- and post- bronchodilator spirometry using the NDD EasyOne Spirometer (Zurich, Switzerland) according to the American Thoracic Society criteria [19]. Pre-bronchodilator spirometry was followed by administration of two puffs of albuterol HFA using appropriate spacers such as Aerochamber® (Monaghan Medical Corporation, Plattsburgh, NY). Post-bronchodilator spirometry was performed 15–20 min post albuterol administration.

### Quantitative CT analysis

Quantitative CT analysis was performed by VIDA (Coralville, IA) [20] and Imbio LLC (Minneapolis, MN) software programs [13]. VIDA was used to assess the percent of low attenuation area below -950HU at full inspiration (%LAA_-950insp_), below -856HU at end-tidal expiration (%LAA_-856exp_), and the average wall thickness of bronchi with 10 mm internal perimeter (AWTPi10). Imbio was used to derive persistent low-density areas (%pLDA) and functional low-density areas (%fLDA) from co-registered inspiratory-expiratory CT scans representing regions of emphysematous and non-emphysematous gas-trapping, respectively.

### Emphysema severity index (ESI)

The ESI software is designed to perform a fast fitting to the descending limb of the MEFV curve suitable for real-time analysis in clinical practice and for large dataset in clinical and pharmacologic trials (freely available for research at url: https://*www.emphysema.app*). The ESI online app ultimately provides a continuous positive numeric parameter ranging from 0 to 10 after receiving discrete input parameters (PEF, FEF25, FEF50, FEF75, FVC) derived from the MEFV curve obtained at spirometry. The procedure is fully automated and the results are calculated real -time. For theoretical background of ESI see reference [18].

### Data analysis

Association between ESI, FEV_1_, FEV_1_/FVC and CT metrics was assessed by Pearson’s r correlation and R^2^ determination coefficients. Robust Steiger’s Z-test was used to assess statistical significance of the difference between correlations [21]. Multiple regression analysis was performed using ESI as dependent variable and %pLDA and %fLDA as independent variables to further evaluate the association profile between the three parameters. Subjects were allocated in subgroups based on the %LAA_-950insp_ cut-off reported by the Fleischner Society for the presence and the severity of emphysema: no emphysema (NE, %LAA_-950insp_ < 6), moderate emphysema (ME, 6 ≤ %LAA_-950insp_ < 14), and severe emphysema (SE, %LAA_-950insp_ ≥ 14) [7, 22]. Differences in ESI among GOLD stages and CT emphysema severity subgroups were assessed by one-way ANOVA and Welch’s *t*-test. The distribution of CT subgroups (NE, ME, SE) within the ESI value range from 0 to 10 was evaluated by contingency table. Goodman and Kruskal’s gamma test was performed to assess the strength of the association between the different ranges of ESI and emphysema CT metrics.

All analyses were performed using SPSS (PCWIN 11.5.1, Chicago, IL, USA) and Orange software [23]. Two-sided alpha 0.05 was considered significant.

## Results

From the 10,371 subjects enrolled in the COPDGene study we excluded those with MEFV curves not satisfying the standard quality criteria (*n* = 1397) or the automated quality check by ESI program (*n* = 71), those with spirometry and CT obtained in a different day (*n* = 1673), those who had spirometry but no CT (*n* = 276), those who had CT analyzed by VIDA software only (*n* = 945), and never smokers (*n* = 79). ESI was calculated in 5930 smokers distributed across all GOLD stages (GOLD 0, *n* = 2446; GOLD 1, *n* = 499; GOLD 2, *n* = 1169; GOLD 3, *n* = 662; GOLD 4, *n* = 313) and PRISm (*n* = 641). Table 1 describes the anthropometric, pulmonary function and CT metrics data of the final population.

**Table 1:** Anthropometric, pulmonary function and CT metrics data of the 5930 subjects of the COPDGene population included in the study

Men/Women Non-Hispanic Whites/African Americans	3128/2802 4389/1541
PRISm/GOLD0/GOLD1–4	641/2646/2643
Age (yr)	60.1 (8.9)
BMI (kg/m^2^)	28.8 (6.2)
Smoking history (pack/years)	44.2 (24.6)
FEV_1_ (%pred)	77.4 (25.0)
FVC (%pred)	87.9 (17.8)
FEV_1_/FVC	0.66 (0.16)
PEF (L/s)	6.4 (2.4)
FEF25% (L/s)	4.8 (2.6)
FEF50% (L/s)	2.5 (1.7)
FEF75% (L/s)	0.6 (0.5)
FVC (L)	3.4 (1.0)
AWTPi10^a^	3.7 (0.1)
%LAA_-950insp_^a^	6.5 (9.7)
%LAA_-856exp_^a^	21.6 (19.5)
%pLDA^b^	4.5 (9.3)
%fLDA^b^	19.5 (14.2)

Data are expressed as mean (SD) or absolute numbers. *AWTPi10 =* Average wall thickness of bronchi with an internal perimeter of 10 mm, *BMI =* Body mass index, *FEF =* Forced expiratory flow, *FEV*_1_% = forced expiratory volume in 1 sec, %fLDA = percentage of functional low density area, *FVC =* Forced vital capacity, *GOLD =* Global initiative for chronic Obstructive Lung Disease; %LAA_-950insp_ = percentage of lung attenuation area with values <− 950 Hounsfield Units at inspiratory CT scan, %LAA_-856exp_ = percentage of lung attenuation area with values <− 856 Hounsfield Units at expiratory CT scan, *PEF =* Peak expiratory flow, %pLDA = percentage of persistent low density area, %pred = percentage of predicted, *PRISm =* Preserved Ratio Impaired Spirometry, i.e. FEV_1_/FVC ≥ 0.7 and post-bronchodilator FEV_1_ < 80% of predicted; ^a^ parameters calculated by using VIDA software; ^b^ parameters calculated by using Imbio software

The correlation and the determination coefficients between functional parameters and CT metrics are reported in Table 2. ESI had stronger correlations with emphysema CT metrics (%LAA_-950insp_, %pLDA) than standard functional parameters of airflow obstruction (FEV_1_, FEV_1_/FVC). ESI had weaker correlations with airway disease (AWTPi10, %fLDA) than with emphysema CT metrics (%LAA_-950insp_, %pLDA). Considering coefficients of determination (R^2^), ESI accounted for 35% of %fLDA variability while explaining roughly the double (67%) of that of %pLDA. At variance with ESI, both FEV_1_ and FEV_1_/FVC had similar R^2^ values for emphysema and airway disease CT metrics. Multiple regression analysis demonstrated that the coefficient of determination R^2^ had only a small increase (from 0.67 to 0.71) of predictive ability when adding %fLDA to the univariate correlation of ESI with %pLDA. This indicates the specificity of ESI in detecting the emphysematous component of whole airway obstruction.

**Table 2:** Pearson’s r correlations and determination coefficients (R^2^) between continuous CT metrics of emphysema (%LAA_-950insp_ and %pLDA) or airway disease (% fLDA and AWTPi10) and functional parameters

	ESI			FEV_**1**_			FEV_**1**_/FVC		
	r	***R***^***2***^	***p***	r	***R***^***2***^	***p***	r	***R***^***2***^	***p***
%LAA_-950insp_^a^	0.80	*0.64*	*<.001*	−0.60	*0.36*	*<.001*	−0.75	*0.56*	*<.001*
%pLDA^b^	0.82	*0.67*	*<.001*	−0.63	*0.40*	*<.001*	−0.74	*0.55*	*<.001*
%fLDA^b^	0.59	*0.35*	*<.001*	−0.58	*0.34*	*<.001*	−0.71	*0.50*	*<.001*
AWTPi10^a^	0.17	*0.03*	*<.001*	−0.34	*0.12*	*<.001*	−0.16	*0.03*	*<.001*

Legend: *AWTPi10 =* Average wall thickness of bronchi with an internal perimeter of 10 mm, %fLDA = percentage of functional low density area, %LAA_-950insp_ = percentage of lung attenuation area with values <−950 Hounsfield Units at inspiratory CT scan, %pLDA = percentage of persistent low density area, ^a^ parameters calculated by using Apollo (VIDA), ^b^ parameters calculated by using Imbio

Table 3 shows differences in ESI values across GOLD stages and emphysema severity groups. All GOLD groups differed significantly for ESI mean values (Welch’s *t-* test *p* < 0.001), increasing in parallel with GOLD stage with high variability within each stage. ESI mean values of both PRISm and GOLD 0 groups (0.9) were lower than those of GOLD 1–4 groups. Likewise, all CT emphysema severity groups differed significantly for ESI mean values (Welch’s *t*-test p < 0.001).

**Table 3:** Analysis of variance for ESI values among PRISm and GOLD stages and for subgroups with different emphysema severity (%LAA_-950insp_) at CT

		N	ESI mean (SD)	ANOVA / Welch’s ***t***-test
PRISm and GOLD stages	PRISm	641	0.9 (0.4)	*p < .001*
	0	2646	0.9 (0.3)	
	1	499	1.4 (0.4)	
	2	1169	2.1 (1.1)	
	3	662	5.0 (2.1)	
	4	313	8.3 (2.0)	
%LAA_-950insp_	No Emphysema (< 6%)	4223	1.1 (0.8)	*p < .001*
	Moderate Emphysema (6–14%)	866	2.4 (2.0)	
	Severe Emphysema (≥14%)	841	5.9 (2.8)	

**Fig. 1:**
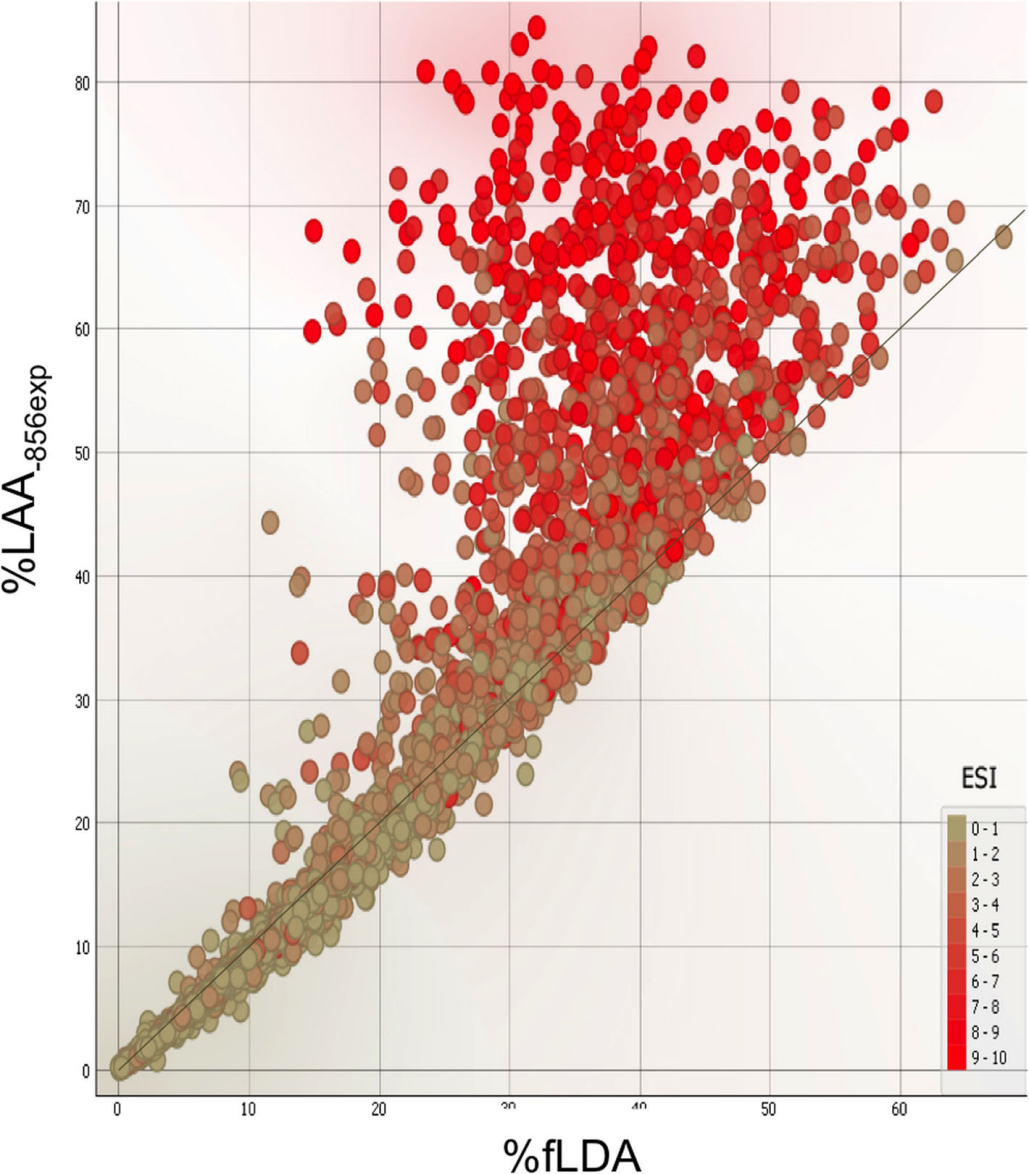
Distribution of the COPDGene population according to CT derived metrics: total expiratory gas trapping (y-axis, %LAA_-856exp_) and functional gas trapping (x-axis, %fLDA). Data points are colored according to the corresponding ESI values calculated by spirometry (progressive range 0–10). Total expiratory gas trapping entails the air trapped in the lungs at the end of a forced expiration due to both conductive airway disease (functional gas trapping, %fLDA) and emphysematous parenchymal destruction, whose amount is represented by the ESI values

Figure 1 shows the color-coded distribution of ESI values (0–10) in a graph comparing functional gas trapping (%fLDA, non-emphysematous gas trapping) with total gas trapping (emphysematous and non-emphysematous, as derived from the relative lung area with CT attenuation below -856HU at expiration). Most ESI values compatible with lower degrees of emphysema were located around the identity line between the two variables. Below 25–30% of total expiratory gas trapping there were only few data points compatible with significant emphysema, indicating that total gas trapping below that level was apparently due only to functional gas trapping. ESI values compatible with greater levels of emphysema were mostly located above 30% of total gas trapping and progressively dispersed above the identity line with the increase in total gas trapping.

**Fig. 2:**
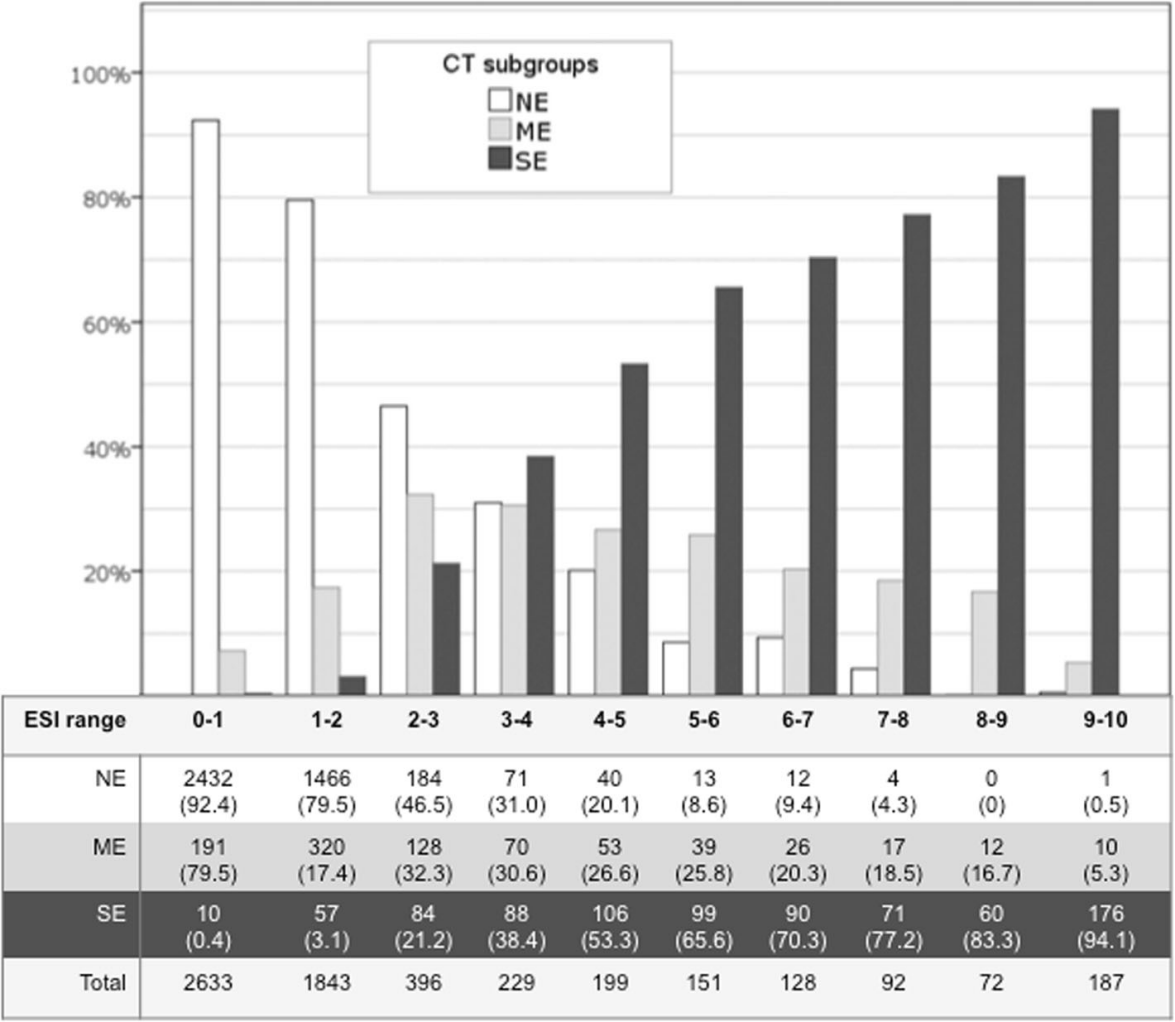
Distribution of ESI values across the different emphysema subgroups as assessed by CT. White bars represent the percentages for NE (no emphysema, %LAA_-950insp_ < 6), light gray bars for ME (moderate emphysema, 6 ≤ %LAA_-950insp_ < 14), and dark gray bars for SE (severe emphysema, %LAA_-950insp_ ≥ 14). Data in the table represent absolute numbers (percentages). The Goodman and Kruskal’s Gamma for the corresponding contingency table is G = 0.82, *p* < 0.001

Figure 2 shows the distribution and the numerical details of the three CT subgroups (NE, ME, SE) among the ranges of ESI values. The Goodman and Kruskal’s gamma for the corresponding contingency table was G = 0.82, *p* < 0.001. Ninety-two percent of subjects with ESI ≤ 1 were classified at CT as NE, 7.3% as ME and 0.4% as SE group. On the other side, 94.1% of subjects with ESI ≥ 9 values were classified at CT as SE, 5.3% as ME and 0.5% as NE. Furthermore, with the progressive increase of ESI values there was a gradual increase in the percentage of subjects classified as SE and a gradual reduction in the percentage of subjects classified as NE. For those classified at CT as ME we observed an increase of the percentage of cases from ESI 0 to 2–3, then a slight trend to reduction.

## Discussion

We have demonstrated the utility of ESI in identifying emphysema in a large and genetically diverse population of current and former smokers. ESI when combined with standard spirometric variables could help in discriminating the prevalent mechanism (i.e. emphysema or airway disease) underlying airflow obstruction. The method relies on each patient’s MEFV curve morphology as derived from absolute values of discrete spirometric variables, thus being independent of percent-predicted values.

The forced expiratory maneuver is the basic lung function test used to detect airflow obstruction [24]. In the late '70s, Saltzman [25] proposed that a “kinking” of the descending limb of the MEFV curve might represent a sign of airway collapse reflecting the presence of emphysema. In the following decades some papers have considered the study of the MEFV curve morphology as a possible method to distinguish normal aging from less paraphysiological airflow obstruction [26] or to predict emphysema [27–29] or chronic bronchitis [30] by spirometry. All studies investigated either the kinking in different portions of the MEFV curve descending limb or its continuous flow-decay (30) using mathematical linear models to approach the curvilinear shape of the MEFV curve.

The shape of the MEFV curve has also been studied to assess whether it could identify mild airflow obstruction in subjects with otherwise normal spirometry [31, 32]. A recent study has shown that the area under the MEFV curve could provide a superior estimation of severe hyperinflation than conventional indices like RV/TLC and IC/TLC in patients with COPD [33]. However, these studies did not differentiate the contribution of emphysema or small airway disease to airflow obstruction. In a previous pilot study, in a small population of patients with COPD, it has been demonstrated that presence and severity of emphysema as quantified by CT metrics and radiomics can be estimated by mathematical modeling of airway function as derived from standard spirometry [18]. Here we confirm in a larger population, including smokers with normal lung function at standard spirometry, that the analysis of MEFV curve descending limb can generate a functional index (ESI) that is more strongly correlated than standard functional parameters with CT metrics indicative of emphysema and to a considerably lesser extent with CT metrics indicating airway disease. At variance with ESI the correlation with emphysema or airway disease CT metrics of standard functional parameters did not significantly differ. These results support the ability of ESI to specifically capture the emphysematous contribution to airflow obstruction. The finding of similar ESI values in PRISm and GOLD 0 groups further support the specificity of ESI in detecting the presence of emphysema. Thus, ESI could complement the assessment of airflow obstruction at spirometry to differentiate patients with similar degrees of airflow obstruction but different degrees of emphysema severity.

Analysis of ESI values across CT subgroups of emphysema severity showed either a significant progressive reduction or increase in ESI values in NE and SE subgroups, respectively. Conversely, ESI values in ME subgroup showed less consistency throughout the CT subgroups. This could possibly be explained by the fact that %LAA_-950insp_ only represents extent of parenchymal destruction as reflected by X-ray attenuation and not an index of specific morphologic features in terms of emphysema subtypes. ME subgroup may then include subjects with mild emphysema, centrilobular or paraseptal. Centrilobular and paraseptal emphysema less than 6% extent on CT can be present also in NE patients (7). This may cause some data dispersion in the correlation between ESI and CT data. In fact, for the same level of %LAA_-950insp_, centrilobular emphysema would affect the MEFV curve morphology to a greater extent than paraseptal emphysema, which is located in the more peripheral regions of the lung. A relationship between the qualitative CT features of the COPD subtypes observed in the COPDGene population [34] and the corresponding ESI values has not been performed yet. The study of this relationship, which is out of the scope of this paper, could be essential to confirm the speculations above.

The distribution of the large COPDGene population according to total and functional gas trapping (Fig. 1) is in line with the previous observation that subjects with an amount of total gas trapping around or below 30% have negligible amounts of emphysema [12]. This suggests that gas trapping in these subjects derives mostly from the non-emphysematous component, in line with recent data showing that terminal bronchioles are narrowed and destroyed before the onset of emphysematous changes [35]. If total gas trapping is around or above 30% the emphysema component adds to the non-emphysematous one, resulting in progressive data dispersion above the identity line. We hypothesize that the severity of emphysema is proportional to the distance from the identity line that relates total gas trapping with functional gas trapping. A longitudinal analysis of COPD progression could ascertain whether the spectrum of the disorder as observed in this study reflects different degrees of severity of the same disease or just different disease entities sharing airflow obstruction at spirometry.

Our study has several strengths. We analyzed a large multiethnic population of smokers who underwent extensive phenotyping with spirometry and CT imaging. Unlike previous studies [27, 28] we compared our results with CT metrics deemed to reflect both emphysema and airways disease. Our method is not a probabilistic predictive model trained on a specific learning set, but it depends only on the specific shape of each MEFV curve. An important step forward is the development of an application for real-time analysis, making ESI suitable for routine clinical use, for application on prospective wide-scale clinical trials, and for application in post-hoc analyses of previous randomized pharmacologic clinical trials to evaluate the effects of emphysema severity on the outcome.

Our study has also limitations. First, the method relies on a well-performed MEFV curve. The automated quality check performed by ESI excluded only 71 patients in whom spirometric data acquisition was closely controlled. The number of patients excluded from the calculation of ESI could be higher in less controlled studies. Second, we were unable to compare our score with the severity of emphysema as assessed by absolute lung volumes and diffusing capacity for carbon monoxide, as these data as well as the measurement of slow vital capacity (VC), were not available in the COPDGene study at baseline. However, in a previous study ESI was strongly correlated with absolute lung volumes and diffusing capacity [18]. Third, we compared ESI with CT metrics that reflect overall parenchymal destruction at predefined attenuation thresholds as surrogate of emphysema. However, the thresholds considered are those generally used in studies comparing CT data with other measurements. Fourth, expiratory CT scans were acquired at end-tidal expiration and not at end-forced expiration that correspond to the end of the expiratory effort at spirometry. However, this difference in lung volumes at expiration could have only reduced our validation performances by CT.

## Conclusions

This study shows that the analysis of the MEFV curve downslope can provide an index of emphysema presence and severity (ESI), independent of percent-predicted values, validated on CT scans and in the large multiethnic population of smokers of the COPDGene study. Therefore, ESI can be applied for clinical and research purposes in the general population of smokers to add to standard spirometry the power to discriminate whether emphysema or airways disease is the predominant mechanism of airway obstruction.

## Data Availability

Individual participant data that underlie the results reported in this article, after de-identification (text, tables, figures, and appendices) will be available together with the study protocol beginning 9 months and ending 12 months following article publication. Data will be available with investigators whose proposed use of the data has been approved by an independent review committee (“learned intermediary”) identified for this purpose. Proposals may be submitted up to 12 months following article publication. After 12 months the data will be available in our university’s data warehouse but without investigator support other than deposited metadata.

## Abbreviations

AWTPi10: Average wall thickness of medium-sized airways with internal perimeter of 10 mm
COPD: Chronic obstructive pulmonary disease
CT: Computed tomography
ESI: Emphysema severity index
FEF: Forced expiratory flow
FEV_1_: Forced expiratory volume in 1 s
%fLDA: Percent of functional low-density area
FVC: Forced vital capacity
VC: Vital capacity
GOLD: Global initiative for chronic Obstructive Lung Disease
HU: Hounsfield Units
%LAA_-950insp_: Percent lung attenuation area below -950HU at inspiratory CT
MEFV: Maximal expiratory flow-volume
NE: No emphysema
ME: Moderate emphysema
PEF: Peak expiratory flow
%pLDA: Percent of persistent low-density area
PRISm: Preserved Ratio Impaired Spirometry
SE: Severe emphysema
